# Characterization of the microbial community structure in *Candidatus* Liberibacter asiaticus-infected citrus plants treated with antibiotics in the field

**DOI:** 10.1186/1471-2180-13-112

**Published:** 2013-05-23

**Authors:** Muqing Zhang, Charles A Powell, Ying Guo, Lesley Benyon, Yongping Duan

**Affiliations:** 1 Indian River Research and Education Center, IFAS-UF, Fort Pierce, FL 34945, USA; 2 USDA-ARS, US Horticultural Lab, Fort Pierce, FL 34945, USA; 3 State Key Laboratory for Conservation and Utilization of Subtropical Agro-bioresources, Guangxi University, Guangxi 530004, China

## Abstract

**Background:**

Huanglongbing (HLB) is a worldwide devastating disease of citrus. There are no effective control measures for this newly emerging but century-old disease. Previously, we reported a combination of Penicillin G and Streptomycin was effective in eliminating or suppressing the associated bacterium, ‘*Candidatus* Liberibacter asiaticus’ (Las).

**Results:**

Here we report the bacterial composition and community structure in HLB-affected citrus plants during a growing season and while being treated with antibiotic combinations PS (Penicillin G and Streptomycin) and KO (Kasugamycin and Oxytetracycline) using the Phylochip™ G3 array. Both antibiotic treatments resulted in significantly lower Las bacterial titers (*Pr*<0.05) and hybridization scores. Of the 50,000+ available operational taxonomic units (OTUs) on PhyloChip™ G3, 7,028 known OTUs were present in citrus leaf midribs. These OTUs were from 58 phyla, of which five contained 100 or more OTUs, *Proteobacteria* (44.1%)*, Firmicutes* (23.5%), *Actinobacteria* (12.4%), *Bacteroidetes* (6.6%) and *Cyanobacteria* (3.2%). In the antibiotic treated samples, the number of OTUs decreased to a total of 5,599. The over-all bacterial diversity decreased with the antibiotic treatments, as did the abundance of 11 OTUs within *Proteobacteria*, *Firmicutes*, *Bacteroidetes* and *Planctomycetes*. Within the *Proteobacteria*, ten OTUs representing the class ***γ***-proteobacteria increased in abundance after four months of treatment, when the Las bacterium was at its lowest level in the HLB-affected citrus field plants.

**Conclusions:**

Our data revealed that *Proteobacteria* was constantly the dominant bacterial phylum recovered from citrus leaf midribs, with the α-proteobacterial and the γ-proteobacterial classes vying for prevalence. In addition, the level of bacterial diversity found in the leaf midribs of field citrus was greater than previously described. Bacterial cells in close proximity may be able to modify their microenvironment, making the composition of the microbial community an important factor in the ability of Las to cause HLB progression. A low Las level was seen as an annual fluctuation, part of the bacterial population dynamics, and as a response to the antibiotic treatments.

## Background

Huanglongbing (HLB) is one of the most serious diseases of citrus and causes great losses in the citrus industry worldwide. It has been reported that since 2006, HLB has cost Florida’s economy an estimated $3.63 billion in lost revenues and 6,611 jobs by reducing orange juice production [1]. HLB is associated with three species of fastidious and phloem-limited α-*proteobacteria* in the genus ‘*Candidatus* Liberibacter’: ‘*Ca.* Liberibacter asiaticus’ (Las), ‘*Ca.* Liberibacter africanus’, and ‘*Ca.* Liberibacter americanus’ [2], of which Las is the only species in the USA. Although HLB resistant citrus varieties are being developed to combat the disease, it will likely take over 10 years to produce and evaluate these resistant varieties in Florida [3]. Since Florida citrus trees are already infected, it is essential to develop an efficient treatment to combat HLB in the interim. Development of a bactericide or other therapeutic compound would provide an additional tool for the control of HLB.

The microbial communities of leaves are diverse and bacteria, of many genera, are the most abundant inhabitants. It is thought that cell density-dependent signaling may play a role in epiphytic bacterial behavior and that cell-cell signaling may influence bacterial fitness [4]. Thus, bacterial cells within aggregates or in close proximity may be able to modify their microenvironment by triggering neighboring bacteria to express traits for their benefit. Therefore, the composition of the microbial community may be important for the ability of Las to cause HLB progression. Certain citrus plants within heavily Las-infected groves appear to “escape” the disease and remain healthy. It has been hypothesized that these plants, which share a similar growing environment, may have a unique microbial composition [5], indicating that the microbial community in citrus may play a key role in the development of HLB. Few reports have described the composition of the bacterial community associated with citrus [5,6], the effects of the season, or the impact of antibiotic treatments on the microbial communities *in planta.* Thus, the dynamics of the citrus bacterial population are not well characterized.

The introduction of antibiotics for the treatment of bacterial diseases revolutionized human medicine. Since then, plant pathologists have been interested in their efficacy for controlling plant bacterial diseases. Antibiotics have been used to control bacterial diseases of fruit trees and to limit contamination in micropropagation and plant tissue culturing for over 50 years [7-9]. Nearly 40 antibiotics have been tested for plant disease control but less than 10 have been used commercially and, of those, only streptomycin and tetracycline have had significant usage in fruit trees [10]. During the 1970s, tetracycline was evaluated by direct injection into the trunks of HLB-affected citrus trees in South Africa, China, and Indonesia [11-14]. However, this practice was discontinued due to labor costs and phytotoxicity. HLB has also previously been controlled by penicillin carbendazin [15,16]. In an earlier study [17], the combination of penicillin and streptomycin was found to be effective in eliminating or suppressing the Las bacterium, and the combination provided a therapeutically effective level of control for a much longer time than when either antibiotic was administered separately.

To increase the throughput of bacterial detection, 16S rRNA gene-based phylogenetic analysis has been commonly employed to characterize microbial diversity [18,19]. A high-density 16S rRNA gene oligonucleotide microarray, the PhyloChip™, has recently been developed and effectively used to study bacterial population diversity. It is particularly adept at identifying bacteria in the environment [20], and a recent study on the bacterial diversity in HLB-affected citrus used the PhyloChip™ G2 and 16S rRNA gene cloned libraries [5]. The updated PhyloChip™ generation 3 (G3) includes 1.1 million probes, the inclusion of strain specific probe sets, the ability to detect over 50,000 operational taxonomic units (OTUs), and over 320,000 sequences in the reference database, which is over 10 times greater than that for the PhyloChip™ G2 [21]. Here, we used the PhyloChip™ G3 array to explore the differences in the relative abundance and phylogenetic diversity of the bacterial communities associated with HLB-affected citrus plants in the field over a growing season and those treated with antibiotic combinations.

## Results

### Dynamic variations of the bacterial community in HLB-affected field citrus

The most prevalent bacterial phylum in citrus leaves in October 2010 was *Proteobacteria* with an average of 1,301 OTUs out of 2,948 OTUs (44.1%). The next most prevalent phylums were the *Firmicutes* (566 of 2,948; 19.2%) and the *Actinobacteria* (458 of 2,948; 15.5%) (Additional file 1: Table S1). The number of OTUs in the *Bacteriodetes* decreased at a statistically significant level (*Pr*<0.05) between October 2010 and April 2011, and that difference appeared to be concentrated in the class of *Flavobacteria*. While the phylum *Proteobacteria* itself remained at 44% of the bacterial community, the number of OTUs in the α-proteobacterial and β-proteobacterial classes decreased significantly (*Pr*<0.05). Among the α*-proteobacteria*, the orders *Rhizobiales* (*Pr*<0.05) and *Sphingomonadales* (*Pr*<0.01) had decreased OTUs, and among the β*-proteobacteria* the order *Burkholderiales* had decreased OTUs (*Pr*<0.05). While the number of OTUs in the γ*-proteobacteria* as a class increased, they decreased in the order *Pseudomonadales* (*Pr*<0.05). The increase in the γ-proteobacterial class was statistically significant, and the difference appears concentrated in the *Enterobacteriales* (*Pr*<0.05). This was the only member of the bacterial community to show an increase in the number of OTUs in April 2011 over October 2010. The total OTUs for all phyla had dropped to 67% of the October 2010 level.

In the period from April 2011 to October 2011, many of the bacterial phyla that had a decrease in OTUs during the proceeding period began to recover. *Actinobacteria*, *Firmicutes*, and *Spirochaetes* all had increased numbers of OTUs, and as a percentage of total OTUs they had all surpassed their October 2010 levels. *Proteobacteria* was still the most abundant phylum but it represented only 39% of the total OTUs in October 2011. The β-proteobacterial class had significantly more OTUs (*Pr*<0.05) as did the order *Burkholderiales* (*Pr*<0.05). The number of OTUs in the γ-proteobacterial class decreased significantly (*Pr*<0.05), and this difference appears concentrated in the order *Enterobacteriales* (*Pr*<0.05). While the bacterial OTU levels appeared to be trending upward, by October of 2011 the overall abundance of bacteria was still only 72% of the October 2010 level.

### Las bacterium in HLB-affected citrus treated with antibiotic combinations

**Figure 1:**
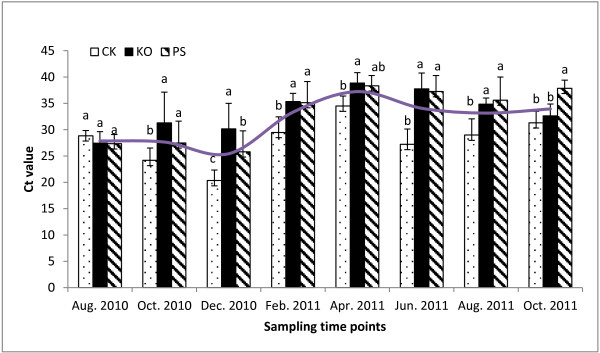
**qPCR Ct values of ‘*Candidatus *Liberibacter asiaticus’ (Las) in Huanglongbing (HLB)-affected citrus treated with antibiotic combinations.** The higher Ct values represent lower Las bacterial titers in the samples. (i) Severe HLB-like symptoms with Ct values <26, and Las bacterial titers of more than 770,000 cells per gram plant tissue, (ii) no symptoms with Ct values ≥36.0, and Las bacterial titers of less than 1,060 cells per gram plant tissue. PS: 5 g/tree penicillin G potassium and 0.5 g/tree streptomycin; KO: 2 g/tree oxytetracycline and 1.0 g/tree kasugamycin; and CK: water as control. The different letters on the bars represent the significance at the 0.05% level (*Pr*<0.05). The smooth top line represents the seasonal fluctuation of the Las bacterium.

**Figure 2:**
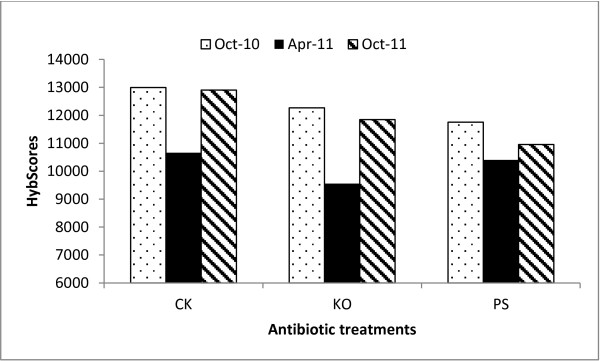
**PhylochipTM HybScores of ‘*Candidatus *Liberibacter asiaticus’ (Las) from Huanglongbing (HLB)-affected citrus.** The citrus plants were treated with antibiotic combinations and sampled at different times (October 2010, April 2011 and October 2011) over a year. (i) Severe HLB-like symptoms with Ct values <26, and Las bacterial titers of more than 770,000 cells per gram plant tissue; (ii) no symptoms with Ct values ≥36.0, and Las bacterial titers of less than 1,060 cells per gram plant tissue. A HybScore change of 1000 indicated a doubling in the fluorescence intensity of the OTU. PS: 5 g/tree penicillin G potassium and 0.5 g/tree streptomycin; KO: 2 g/tree oxytetracycline and 1.0 g/tree kasugamycin; and CK: water as control.

The dynamic variations of Las bacterial titers from August 2010 to October 2011 at the USHRL farm, Fort Pierce, FL are presented in Figure 1. The results showed that the Las bacterial population fluctuated throughout the year in HLB-affected citrus plants with or without antibiotic treatments. The highest Las bacterial titers (lowest Ct values) were observed in December 2010, and the lowest Las bacterial titers (highest Ct values) were recorded in April 2011. This variation generally coincided with HLB-symptoms in the field. There were no significant differences among the antibiotic treatments and the water control before the initial applications in August 2010. Two months after the initial applications, significant differences (*Pr*<0.05) existed between the antibiotic treatments and the controls. By April 2011, the titers had decreased by more than 13-fold in the water control, 259-fold in the KO treated citrus and 97-fold in the PS treated citrus. The HybScore of OTU63806, which represented *Candidatus* Liberibacter from PhyloChip™ G3, coincided with the Las bacterial titers detected by qPCR (r=0.812). HybScores averaged 12,186±1,320 in the untreated trees (water control, CK) compared to 11,226±1,458 and 11,037±678 in the HLB-affected trees treated with KO and PS, respectively. HybScores were the lowest in April 2011 when the HLB-bacterial population was also at its lowest level (Figure 2).

### Bacterial community structure and diversity

The PhyloChip™ G3 array was used to gain insights into the structural composition and diversity of bacteria in the leaf midrib from HLB-affected citrus treated with antibiotic combinations (PS and KO). Of the 7,028 OTUs from our field citrus samples found on the PhyloChip™ G3, a total of 5,599 (79.7%) were detected in our antibiotic treated field samples. The number of OTUs found per treatment (PS, KO or CK) and sampling time point (October 2010, April 2011 or October 2011) ranged from 1,981 to 2,487 (Additional file 1: Table S1). In total, 58 phyla were detected, of which five phyla had 100 or more OTUs, *Proteobacteria* (3,099 proteobacterial OTUs out of 7,028 total OTUs, 44.1%)*, Firmicutes* (1,651 of 7,028 OTUs, 23.5%), *Actinobacteria* (874 of 7,028 OTUs, 12.4%), *Bacteroidetes* (466 of 7,028 OTUs, 6.6%) and *Cyanobacteria* (222 of 7,028 OTUs, 3.2%). *Proteobacteria* were still dominant in the bacterial populations after treatments. In trees receiving the antibiotic combinations KO and PS, the average OTUs over sampling time points accounted for 44.5% and 44.2%, respectively, of the treated populations, while they represented 38.9% of the control population. *Proteobacteria* were also dominant in the bacterial population at all sampling time points. The average OTUs in the antibiotic treatments accounted for 44.1%, 43.9% and 38.6% of the bacterial population in October 2010, April 2011, and October 2011, respectively. When compared to the bacterial populations in the leaves of trees receiving the water control treatment, the *Bacteroidete* population decreased (*Pr*<0.05) by 65.3% and 51.8% in the leaves of trees receiving the KO and PS treatments, respectively (Additional file 1: Table S1).

**Figure 3:**
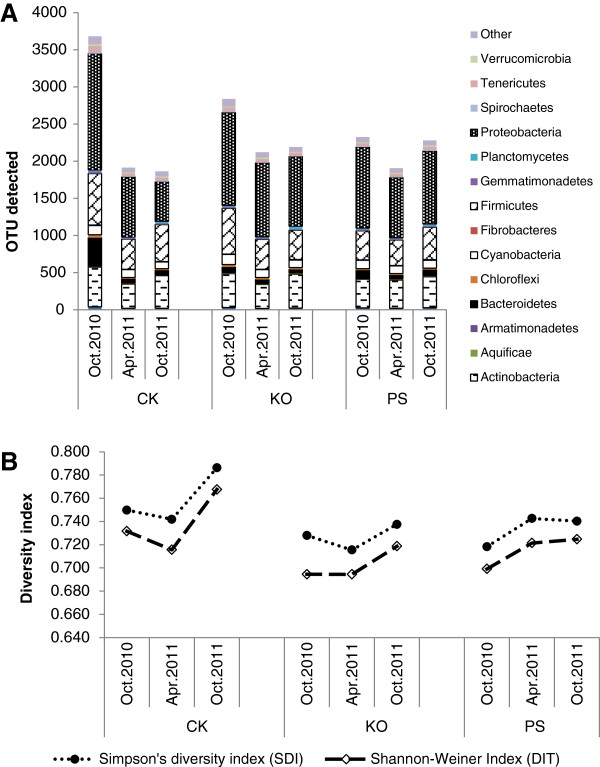
**Bacterial richness and diversity in phyla detected by PhyloChip™ G3 hybridization of Huanglongbing (HLB)-affected citrus.** The citrus plants were treated with different antibiotic combinations, and leaf samples were collected at different times (October 2010, April 2011 and October 2011) over a year. **A**, Total operational taxonomic units (OTUs) in each treatment; **B**, Simpson’s diversity index (SDI) and Shannon-Weiner index (DIT). Each bar represents the coded relative abundance of bacteria in a single phylum. For each treatment, the Simpson’s and Shannon’s diversity statistics, which reflect both species numbers and evenness of species distribution, were plotted below the histogram. PS: 5 g/tree penicillin G potassium and 0.5 g/tree streptomycin; KO: 2 g/tree oxytetracycline and 1.0 g/tree kasugamycin; and CK: water as control.

The PhyloChip data indicated a change in the community profile over the sampling time points and showed fewer unique OTUs in populations subjected to antibiotic treatments (Additional file 1: Table S1; Figure 3A). The lowest number of OTUs was detected in April 2011 after the antibiotics had been applied four times (Additional file 1: Table S1). The phylum *Bacteriodetes*, and specifically the class *Flavobacteria*, significantly decreased (*Pr*<0.05). While the phylum *Proteobacteria* did not decrease, both the classes α*-* and β*-proteobacteria* did decrease significantly (*Pr*<0.05). OTUs within the order of *Rhizobiales* and the family of *Rhizobiaceae* were significantly decreased by the antibiotic treatments. Shannon’s and Simpson’s indices both revealed greater diversity in the water control (Figure 3B), indicating that antibiotic treatments lead to lower phylum diversity.

**Figure 4:**
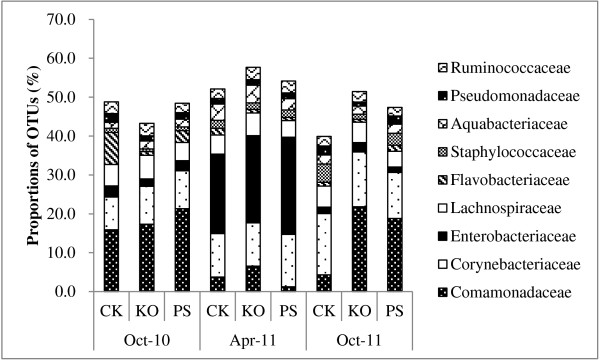
**Operational taxonomic units (OTUs) for families detected by PhyloChip™ G3 hybridization of Huanglongbing (HLB)-affected citrus.** The citrus plants were treated with different antibiotic combinations and leaf samples were collected at different times (October 2010, April 2011 and October 2011) over a year. Proportions of OTUs for the most highly represented families are represented over the sampling time points. The size of each block in the family abundance bar chart represents the number of detected OTUs in that family relative to the total number of OTUs detected with the same treatment over the sampling time points. PS: 5 g/tree penicillin G potassium and 0.5 g/tree streptomycin; KO: 2 g/tree oxytetracycline and 1.0 g/tree kasugamycin; and CK: water as control.

The proportions of OTUs for the most highly represented families (Figure 4) showed a large variation in the number of OTUs detected in the *Comamonadaceae*, *Staphylococcaceae*, *Corynebacteriaceae*, and *Flavobacteriaceae* families. In accordance with the Las bacterial titers, the amount of OTUs in *Comamonadaceae* significantly decreased in April 2011 when compared to the other sampling time points (October 2010 and October 2011); however, the amount of OTUs in the *Enterobacteriaceae* and *Aquabacteriaceae* families significantly increased.

### Specific OTUs associated with the antibiotic treatments and sampling time points

**Figure 5:**
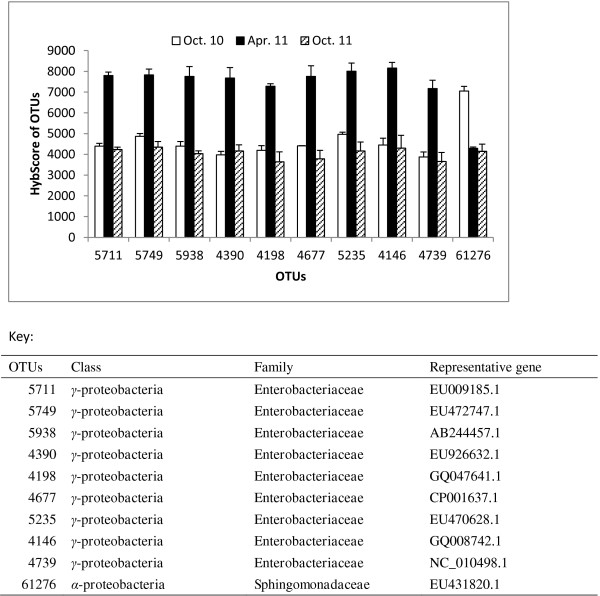
**PhyloChip™ G3 HybScore profiles of operational taxonomic units (OTUs) identified by Prediction Analysis for Microarray (PAM).** Selected OTUs from leaf samples of Huanglongbing (HLB)-affected citrus treated with different antibiotic combinations at different sampling time points. PAM identified nine *Enterobacteriaceae* OTUs (OTUs 5711, 5749, 5938, 4390, 4198, 4677, 5235, 4146 and 4739) with increased abundance levels in the April 2011 samples when the ‘*Candidatus* Liberibacter asiaticus’ (Las) bacterial titers were the lowest compared to samples collected in October of 2010 and 2011, and one *Sphingomonadaceae* OTU, 61276, with an increased abundance level in October 2010.

Principal coordinate analysis (PCoA) based on the weighted Unifrac distances between samples was performed with PhyloChip community data sets, and the results suggested that there were significant differences among the treatments and the sampling time points. The 17 OTUs selected with filter-3, which includes OTUs present in samples from one treatment but not detected in any samples of the other treatments, separated the antibiotic combinations (KO, PS) and the control group (CK). There were eight OTUs (7444, 8217, 15010, 24693, 41872, 62344, 74687 and 77432) in the KO treatment, three in the PS treatment (24114, 40218 and 49638) and six in the water control (42278, 50217, 53352, 58803, 70400 and 75179). When compared with the Antibiotic Resistance Genes Database [22], three oxytetracycline-resistant bacteria (7444, 24693 and 72432) were found in the KO treatment (Table 1). No antibiotic-resistant bacteria were found in the PS treatment. Prediction analysis for microarrays (PAM) identified Bacillus OTU48007 within *Firmicutes* to have increased abundance in the control samples compared to the antibiotic treatments. A total of 118 OTUs with filter-5, based on abundance metrics, partitioned the samples into distinct groups corresponding to sampling time points. Using binary metrics, 344 OTUs selected with filter-5 were found in 100% of the samples from one time point and were consistently absent in other time point samples. PAM also identified nine γ-proteobacterial OTUs (4146, 4198, 4390, 4677, 4739, 5235, 5711, 5749 and 5938) with increased abundance levels in April 2011 samples compared to samples collected in October 2010 and October 2011, and one *Sphingomonadaceae*, OTU61276, with an increased abundance level in October 2010 (Figure 5).

**Table 1:** Specific operational taxonomic units (OTUs) detected at all time points in each antibiotic treatment, KO and PS, in the midribs of leaves from Huanglongbing-affected citrus

**Antibiotic treatments**	**Specific OTUs**	**Representative gene**	**Genus**	**Antibiotic-resistant bacterium **^**Z**^
KO	15010	EF562200.1	Ralstonia	
	8217	GQ091863.1	Diaphorobacter	
	72432	EU455875.1	Lactobacillus	Oxy-resistant bacteria
	41872	AB211018.1	Thermobifida	
	62344	AB473971.1	unclassified	
	24693	DQ798754.1	Faecalibacterium	Oxy-resistant bacteria
	74687	U24588.1	sfA	
	7444	NC006370.1	Photobacterium	Oxy-resistant bacteria
PS	24114	EU456745.1	unclassified	
	49638	FN356252.1	unclassified	
	40218	FJ152555.1	Isoptericola	
CK	75179	AB177144.1	unclassified	
	53352	EU381839.1	Fibrobacter	
	70400	FJ374203.1	unclassified	
	42278	AY660689.1	unclassified	
	58803	AB486305.1	sfA	
	50217	GQ101329.1	Veillonella	

KO: 2 g of oxytetracycline + 1.0 g of kasugamycin per tree.

PS: 5 g of penicillin G potassium + 0.5 g of streptomycin per tree.

CK: water control.

^Z^ Listed in the ARGD (Antibiotic Resistance Genes Database).

## Discussion

The high-density 16S rRNA gene oligonucleotide microarray, the PhyloChip™, is employed to study bacterial population diversity, and it is effective for identifying bacteria in the environment [5,23]. The PhyloChip™ G3 array used in this study contains over 50,000 OTUs representing all demarcated bacterial and archaeal orders [21]. Our results revealed the presence of a total of 7,028 bacterial OTUs in 58 phyla for the field citrus leaf midribs, but no *archaea* were detected in any of the samples.

The bacterial population of citrus leaves on trees that are asymptomatic for HLB includes *Planctomycetes*, *Verrucomicrobia*, *Proteobacteria*, *Actinobacteria*, *BRC1*, *Chlamydiae*, *Chlorobi* and *Acidobacteria *[5], with *Proteobacteria* being the dominant phylum. In addition to the above mentioned bacteria, other bacteria, including *Bacteroidetes* and *Chloroflexi*, have been found in one citrus grove but not in a second grove [5]. Thus, the site appears to influence the composition of the microbial community. In leaves of the evergreen Southern Magnolia tree *Proteobacteria* were also the most populous bacteria, accounting for 53-80% of identified 16S rRNA gene sequences, followed by *Bacteriodetes* (11-38%), *Acidobacteria* and *Actinobacteria *[24]. The month of sampling significantly influenced the phylogenetic compositions of the bacterial population, indicating a seasonal fluctuation in bacterial communities [24]. Seasonal variations in the epiphytic populations of bacteria have also been documented in the olive [25]. Thus, there appears to be both spatial and temporal variations in leaf microbial communities.

Citrus leaves can support a variety of microbes. The PhyloChip™ analysis in a previous study discovered 47 orders of bacteria in 15 phyla [5]. In our study, 58 phyla were revealed using the Phylochip™ G3 array. However, the seasonal variation in the microbial population of citrus has not been extensively studied. The annual fluctuation of endophytic bacteria in Citrus Variegated Chlorosis (CVC) affected citrus showed significant seasonal variations. Yet, as in our study, *Proteobacteria* was constantly the dominant phylum of bacteria recovered with the α-proteobacterial and the γ-proteobacterial class vying for prevalence. The α-proteobacterial class’ *Methylobacterium* spp. was the most populous at three (March-April 1997; September-October 1997; March-April 1998) of the four time points and the γ-proteobacterial class’ *P. agglomerans* was the most populous at the final time point (September-October 1998) [26].

The bacterial diversity of HLB-affected citrus leaves was analyzed only once previously using the PhyloChip™ G2. The bacterial community included *Proteobacteria* (47.1%), *Bacteroidetes* (14.1%), *Actinobacteria* (0.3%), *Chlamydiae* (0.2%), *Firmicutes* (0.1%), *TM7* (0.05%), *Verrucomicrobia* (0.05%), and *Dictyoglomi* (0.01%) [5]. In the present study, we also identified *Proteobacteria* (38.9%), *Actinobacteria* (17.4%), *Bacteroidetes* (6.8%), *Verrucomicrobia* (0.64%), and *Firmicutes* (21.4%); however, we identified several other phyla (Figure 3A). In the former study the community structure was different between the two groves analyzed; thus, our results from a separate location are not atypical.

Prediction analysis for microarrays (PAM) identified ten γ-proteobacterial OTUs (4146, 4198, 4288, 4390, 4677, 5165, 5711, 5938, 6090 and 6095) with increased abundance levels in the April 2011 samples compared to samples collected in October of 2010 and 2011. The abundance of these OTUs appears to be seasonally driven since there is no statistical difference between samples receiving the water control and the antibiotic treatments. These are all members of the large *Enterobacteriaceae* family of Gram-negative bacteria. Some members of this family produce endotoxins that reside in the cell cytoplasm and are released upon cell death with the disintegration of the cell wall. The roles of these endophytic bacteria in HLB development remains to be investigated.

To understand the role of Las in HLB progression, it may be important to separate the temporal changes in the microbial community from the changes caused by or associated with HLB. These seasonal changes have been seen not just in the surface phyllosphere but also in the internal tissues as well. Bacterial populations in the xylem undergo temporal variations in shade trees [27]. In grape vines it has been shown that the endophytic community is similar in healthy plants and plants with undetectable levels of phytoplasmas, but it is different in recovered plants [28]. This reorganization of the bacterial community could indicate direct competition between the infective agent and the endophytic bacteria. It could also be the effect of the plant defense response selecting different strains to adapt to new niches. In addition, the modification of the quantitative levels of some bacteria by the infection could alter the relative bacterial proportions. After antibiotic treatments, *Proteobacteria, Firmicutes*, *Actinobacteria*, *Bacteroidetes* and *Cyanobacteria* were dominant in the bacterial populations. The Phylochip™ G3 indicated that the OTU62086, representing “*Candidatus* Liberibacter”, was detected in all treatments, but had a lower HybScore in the antibiotic treatments, which corresponded with the titers of the Las bacterium. In our previous reports [17,29,30], penicillin alone and its combinations with streptomycin were effective in eliminating or suppressing the Las bacterium in greenhouse plants. In this research, trunk-injections of the antibiotic combinations of penicillin and streptomycin, or kasugamycin and oxytetracycline, suppressed the Las bacterium in HLB-affected citrus in the field throughout the growing season. Las bacterial titers were significantly lower in the PS- or KO-treated HLB-affected trees compared to untreated trees (water control) two months after the initial applications in August 2010 (*Pr*<0.05). The Las bacterial titers increased in the KO-treatment, but remained at a significantly lower level in the PS-treated trees (*Pr*<0.05) for two months (October 2011) after the antibiotic treatments ceased in August 2011. A graft-based chemotherapy analysis of streptomycin and kasugamycin, two amnioglycoside antibiotics, revealed that they were not very effective in suppressing the Las bacterium when each antibiotic was applied alone (data not shown). The effectiveness of penicillin or oxytetracycline against the Las bacterium was enhanced due to the use of antibiotic combinations [30]. Because tetracycline is bacteriostatic rather than bactericidal, it is necessary to frequently apply oxytetracycline for continuous suppression of HLB [15,31]. Thus, it is important to use the antibiotics in combination to decrease the emergence of antibiotic resistant bacteria and to improve the efficacy against the bacteria [32]. In this experiment three OTUs were identified, by searching the Antibiotic Resistance Genes Database [22], as oxytetracycline resistant genes but no penicillin resistant genes emerged. This research may assist regulatory agencies in evaluating the potential for applying antibiotic treatments in the future to larger grove settings.

The temporal and antibiotic-dependent significant differences in the bacterial community have implications not only for biocontrol but also for phytopathology. The composition of the bacterial community may strongly influence the establishment of antagonistic bacteria at appropriate times during plant development or the growing season. By understanding the composition of, and variation in, the bacterial community of citrus we may be able to time HLB control treatments better and to harness the plants own natural microbial population. This will help establish better management and treatment strategies.

## Conclusions

Using the Phylochip™ G3 array, the bacterial composition and community structure in HLB-affected citrus plants during a growing season and while being treated with antibiotic combinations PS and KO were studied. We identified *Proteobacteria* as the major phylum in citrus leaf midribs from the USHRL farm in Fort Pierce, FL. While *Proteobacteria* were the dominant bacteria throughout the growing season, the α-proteobacterial and β-proteobacterial classes decreased significantly (*Pr*<0.05) from October 2010 to April 2011 and the γ*-proteobacteria* as a class increased (*Pr*<0.05). From April 2011 to October 2011 the β-proteobacterial class had significantly more OTUs (*Pr*<0.05) and the number of OTUs in the γ-proteobacterial class had decreased significantly (*Pr*<0.05). These temporal fluctuations in the bacterial population may affect the microenvironment; thus, making the composition of the microbial community an important factor in the ability of Las to cause HLB progression. Both antibiotic treatments, PS and KO, resulted in decreases in the number of OTUs in the dominant phyla, except *Cyanobacteria*, and the over-all diversity of bacteria decreased from 7,028 OTUs to 5,599 OTUs by April 2011. The antibiotic treatments resulted in significantly lower Las bacterial titers (*Pr*<0.05) and hybridization scores. However, within the *Proteobacteria*, ten OTUs representing the class γ*-proteobacteria* increased in abundance after four months of treatment, when the Las bacterium was at its lowest level in the HLB-affected citrus field plants. Antibiotics altered the taxonomic composition of the bacterial community and reduced their diversity while suppressing the Las bacterium. Our data revealed that Las levels fluctuated temporally, as part of the over-all bacterial population dynamics, and as a response to the antibiotic treatments.

## Methods

### Antibiotic treatments on HLB-affected citrus

The antibiotic treatments were conducted in a randomized complete block design with four replicates. For each replicate, five HLB-affected, 7-year-old citrus trees (a unique hybrid, 10c-5-58, which is an open-pollinated seedling from the combination of Lee mandarin × Orlando tangelo) at the USHRL farm, 10 cm in diameter, were injected with either 100 ml of the antibiotic combination treatment PS (5 g of penicillin G potassium + 0.5 g of Streptomycin per tree) or the antibiotic treatment KO (2 g of oxytetracycline + 1.0 g of kasugamycin per tree). Five trees were injected with water as injection controls (CK). Injections were made using an Avo-Ject syringe injector (a catheter-tipped 60 ml syringe; Aongatete Coolstores Ltd., NZ) beginning in August of 2010. The tapered tip was firmly fitted into a 19/64-in (7.5 mm) diameter hole, ≈3 cm deep, drilled into the tree. The injector was kept in the tree and the treatment lasted for one week in each injection-trunk. Treatments were repeated every two months for one year and ceased in August of 2011. Before and during treatment more than 30 leaf samples per tree were taken from different positions around the tree canopies for qPCR assays at two month intervals.

### Genomic DNA extraction and qPCR analysis for the HLB bacterium

Each leaf sample was rinsed three times with sterile water. Midribs were separated from the leaf samples and cut into pieces of 1.0 to 2.0 mm. DNA was extracted from 0.1 g of tissue (fresh weight) of leaf midribs using Qiagen’s DNeasy Plant Mini Kit (Qiagen, Valencia, CA) according to the manufacturer’s protocol. The bacterial titers were quantified by qPCR using the primers and probes (HLBas, HLBr, and HLBp) for ‘*Ca.* L. asiaticus’ as described previously [17,33]. Data were analyzed by a generalized linear mixed model using the SAS procedure GLIMMIX. Differences among treatments and sampling time points were determined with the LINES option of the LSMEANS statement.

### PCR amplification of 16S rRNA genes for PhyloChip™ G3 hybridization

DNA for the PhyloChip™ G3 analysis, which was extracted from 20 samples of the same treatment, was pooled in equal amounts and quantified by the *PicoGreen®* method. The PhyloChip™ G3 analysis was conducted by Second Genome Inc. (San Francisco, CA). The bacterial 16S rRNA genes were amplified from the above pooled DNA using an eight-temperature gradient PCR (annealing temperatures of 48.0, 48.8, 50.1, 51.9, 54.4, 56.3, 57.5, and 58.0°C) with bacterially directed primers 27 F (5-AGA GTT TGA TCC TGGCTC AG) and 1492R (5-GGT TAC CTT GTT ACG ACT T). In brief, the 25 μl reactions (final concentrations were 1× Ex Taq Buffer with 2 mM MgCl_2_, 200 nM each primer (27 F and 1492R), 200 μM each dNTP, 25 μg bovine serum albumin (Roche Applied Science, Indianapolis, IN), and 0.625 U Ex Taq (TaKaRa Bio Inc., Shiga, Japan) were amplified using an iCycler (Bio-Rad, Hercules, CA) under the following thermocycling conditions: 95°C for 3 min for initial denaturation, 35 cycles of 95°C for 30 s, 48 to 58°C for 30 s, and 72°C for 2 min, and then final extension for 10 min at 72°C. PCR products from each annealing temperature for a sample were combined and concentrated using Amicon centrifugal filter units (Millipore Corp., Billerica, MA). The samples were quantified by electrophoresis using an Agilent 2100 Bioanalyzer® before application to the PhyloChip™ G3 array. PhyloChip Control Mix™ was added to each amplified product.

### PhyloChip™ G3 hybridization

About 500 ng of purified PCR product (amplicons) was applied to each PhyloChip™ G3 following the described procedures [21]. Briefly, the 16S rRNA amplicons and a mixture of amplicons at known concentrations were combined, fragmented using DNAseI (Invitrogen, Carlsbad, CA), and biotin-labeled using the recommended protocol for Affymetrix Prokaryotic Arrays. Labeled products were hybridized overnight at 48°C and 60 rpm. The arrays were washed, stained, and scanned as described in Hazen et al. [21].

### Data collection and analysis

Details on probe selection, probe scoring, data acquisition, and preliminary data analysis are presented in Hazen et al. [21] and the analyses were performed by Second Genome (San Bruno, CA, USA). In brief, two criteria were met when the probe pairs scored as positive: (i) the PM (Perfect Match) probe’s intensity of fluorescence was greater than 1.3 times that from the MM (Mismatch) control and (ii) the difference in intensity, PM minus MM, was at least 500 times greater than the squared noise value (>500 *N*^2^), which was the variation in pixel intensity signals observed by the scanner as it read the array surface. An OTU was considered present in the sample when over 90% of its assigned probe pairs were positive. A hybridization intensity score (HybScore) was calculated in arbitrary units for each probe set as the trimmed average (maximum and minimum values removed before averaging) of the PM minus MM intensity differences across the probe pairs in a given probe set. The values of the present OTUs used for each taxa-sample intersection were populated in two distinct ways. In the first case, the abundance metrics were used directly (AT). In the second case, binary metrics were created where 1’s represented presence, 0′s indicated absence (BT). OTUs were filtered in several different manners. Filter-1 includes OTUs present in at least one of the samples. Filter-3 includes OTUs present in samples from one treatment but not detected in any samples of the other treatments. Filter-5 includes OTUs whose abundance significantly increased in one treatment compared to the other treatments and Filter-9 includes OTUs with unique abundance patterns within a species. For Filter-3, the percent prevalence required among the samples in one state began at 100% but then decreased until the OTU set intersected all samples. Thus, each sample contained a present call for at least one of the passing OTUs. The Unifrac distance metric determines the dissimilarity between communities by using the phylogenetic distances between OTUs [34]. For the weighted Unifrac distance metric, WUnifrac, the OTU abundance was also considered. The presence/absence (BT) data, used Unifrac; whereas, the abundance data (AT) used WUnifrac. For Filter-5, p-values were calculated using the parametric Welch test. In this exploratory analysis, false discovery rates were not considered in the p-value calculations. For Filter-9, OTUs were clustered via an average-neighbor (HC-AN) method such that all the OTUs in a group were from the same species and had similar abundance patterns. Abundance patterns were measured by the correlation of abundance vectors across all samples. The max HybScore and min HybScore (the two most variable scores) of OTUs from each treatment were selected and the remaining OTUs were discarded. Principal Coordinate Analysis (PCoA) used the dissimilarity values to position the sample points relative to each other. Significant OTUs, those whose abundance characterized each class, were compiled using Prediction Analysis for Microarrays (PAM) using a nearest shrunken centroid method [35].

### Bacterial biodiversity index

The Shannon and Simpson biodiversity indexes combine both components of species number and their relative abundance [36]. Here they were used to analyze the differences in bacterial diversity among the antibiotic combination treatments calculated from present OTUs as: Shannon’s index, $H\prime =-\sum {}_{1}^{n}P_{i}\;{\mathrm{LnP}}_{i}$, and Simpson’s index, $D=\sum {}_{1}^{n}P_{i}2$. Where n represents the richness or total number of phyla, *P*_*i *_is the proportion of the present OTUs accounted for by the *i*^th ^phylum from the total OTUs detected and *Ln* was the natural logarithm.

## Availability of supporting data

The data sets supporting the results of this article are available in the Geo repository, GSE46727 http://www.ncbi.nlm.nih.gov/geo/query/acc.cgi?acc=GSE46727.

## Competing interests

The authors declare that they have no competing interests.

## Authors’ contributions

MZ, YG and LB carried out the field studies and the DNA extractions. CP and YD participated in the design of the study and its coordination. MZ, LB, YD and CP performed the analysis and drafted the manuscript. All authors read and approved the final manuscript.

## Supplementary Material

Additional file 1: Table S1 Average number of operational taxonomic units (OTUs) detected by PhyloChip™ G3 hybridization in the treatments over the sampling time points and in the sampling time points over the treatments from Huanglongbing (HLB)-affected citrus plants treated with different antibiotic combinations. Table of operational taxonomic units (OTUs) in bacterial phyla based on antibiotic treatments and sampling time points. Click here for file
