# Supplementary material for: Characterization of the microbial community structure in Candidatus Liberibacter asiaticus-infected citrus plants treated with antibiotics in the field
Source: BMC Microbiol. 2013 May 23;13:112. doi: 10.1186/1471-2180-13-112 (PMC3672075; doi:10.1186/1471-2180-13-112)
Supplement: Additional file 1: Table S1 — Average number of operational taxonomic units (OTUs) detected by PhyloChip™ G3 hybridization in the treatments over the sampling time points and in the sampling time points over the treatments from Huanglongbing (HLB)-affected citrus plants treated with different antibiotic combinations. Table of operational taxonomic units (OTUs) in bacterial phyla based on antibiotic treatments and sampling time points. [file 1471-2180-13-112-S1.docx]

Supplemental Table 1. Average number of operational taxonomic units (OTUs) detected by PhyloChip™ G3 hybridization in each treatment from HLB-affected citrus plants treated with different antibiotics (n=3)

| Phylum | Class | Order | | Family | Antibiotic treatment | | | Time points | | | |  | |
| --- | --- | --- | --- | --- | --- | --- | --- | --- | --- | --- | --- | --- | --- |
|  |  |  |  |  | CK | KO | PS | Oct.10 | | Apr.11 | Oct.11 | Total^Z^ | |
| Acidobacteria | |  | |  | 27±12 a | 25±7a | 26±4a | 33±8a | | 20±2a | 24±5a | 86 | |
| Actinobacteria | |  | |  | 433±103a | 411±77a | 393±19a | 458±74a | | 343±31a | 436±20a | 874 | |
| Aquificae | |  | |  | 5±1a | 7±3a | 5±3a | 4±2a | | 6±1a | 7±4a | 15 | |
| Armatimonadetes | | | |  | 7+1a | 7±2a | 7±1a | 8±1a | | 7±1a | 6±1a | 11 | |
| Bacteroidetes | |  | |  | 170±186a | 59±13b | 82±29b | 190±169a | | 56±4b | 65±18b | 466 | |
|  | Bacteroidia | | |  | 30±12a | 21±4a | 22±5a | 29±13a | | 21±2a | 24±6a | 86 | |
|  | Flavobacteria | | |  | 121±163a | 24±7b | 45±30b | 139±149a | | 25±9b | 26±13b | 324 | |
|  | Sphingobacteria | | | | 18±13a | 14±4a | 15±2a | 22±8a | | 10±4a | 15±2a | 56 | |
| Chloroflexi | |  | |  | 25±12a | 29±5a | 21±1a | 31±10a | | 22±5a | 22±3a | 72 | |
| Cyanobacteria | |  | |  | 106±16a | 113±20a | 108±6a | 124±11a | | 104±1a | 99±8a | 222 | |
| Fibrobacteres | |  | |  | 6±2a | 5±2a | 5±1a | 6±2a | | 5±1a | 6±1a | 9 | |
| Firmicutes | |  | |  | 532±145a | 471±126a | 391±48a | 566±156a | | 384±36a | 443±52a | 1,651 | |
|  | Bacilli | | |  | 233±40a | 185±47a | 178±41a | 225±51a | | 165±24a | 206±47a | 678 | |
|  | Clostridia | | |  | 299±109a | 287±79a | 213±11a | 341±105a | | 219±20a | 238±21a | 973 | |
| Gemmatimonadetes | | | |  | 20±7a | 18±3a | 17±2a | 22±6a | | 16±2a | 18±2a | 36 | |
| Planctomycetes | |  | |  | 21±7a | 25±13a | 20±3a | 20±3a | | 15±2a | 31±8a | 70 | |
| Proteobacteria | |  | |  | 967±535 a | 1060±166 a | 959±142 a | 1301±241a | | 870±108a | 815±248a | 3,099 | |
|  | Alphaproteobacteria | | | | 183±121a | 158±91a | 127±57a | 256±67a | | 85±11b | 127±6b | 578 | |
|  |  | Caulobacterales | | | 6±4a | 10±7a | 4±2a | 8±2a | | 3±1a | 10±7a | 12 | |
|  |  | Rhizobiales | | | 83±46a | 66±34a | 51±18a | 102±35a | | 41±12b | 56±3b | 217 | |
|  |  |  | Bradyrhizobiaceae | | 10±4a | 10±1a | 5±2a | 10±5a | | 7±3a | 7±2a | 31 | |
|  |  |  | Hyphomicrobiaceae | | 8±4a | 6±3a | 4±3a | 8±4a | | 3±2a | 7±1a | 19 | |
|  |  |  | Methylocystaceae | | 25±17a | 21±12a | 19±13a | 37±5a | | 13±6b | 15±4b | 53 | |
|  |  |  | Phyllobacteriaceae | | 8±7a | 5±3a | 5±1a | 9±5a | | 3±2a | 5±1a | 24 | |
|  |  |  | Rhizobiaceae | | 16±7a | 11±7a | 8±2a | 17±9a | | 8±3a | 10±3a | 48 | |
|  |  |  | Rhodobiaceae | | 3±2a | 2±2a | 1±1a | 4±2a | | 0±1a | 2±1a | 4 | |
|  |  |  | Other | | 14±9a | 12±5a | 8±3a | 18±6a | | 6±2a | 10±2a | 38 | |
|  |  | Rhodospirillales | | | 48±33a | 35±23a | 28±7a | 59±28a | | 21±1a | 32±7a | 147 | |
|  |  | Rickettsiales | | | 23±14a | 17±3a | 14±4a | 24±13a | | 13±3a | 17±2a | 64 | |
|  |  | Sphingomonadales | | | 20±22a | 27±30a | 29±35a | 59±13A | | 7±1B | 11±2B | 114 | |
|  |  | Other | |  | 3±4a | 2±1a | 1±1a | 4±3a | | 0±1a | 2±1a | 24 | |
|  | Betaproteobacteria | | | | 351±303a | 482±182a | 413±264a | 631±60a | | 189±82b | 426±225ab | 1,581 | |
|  | Burkholderiales | | | | 326±298a | 458±181a | 397±264a | 605±57a | | 169±79b | 408±228ab | 945 | |
|  | Other | | | | 24±4a | 24±5a | 16±3b | 26±6a | | 20±4b | 18±5b | 71 | |
|  | Deltaproteobacteria | | | | 54±30a | 50±20a | 43±4a | 69±20a | | 36±7a | 42±5a | 206 | |
|  | Epsilonproteobacteria | | | | 15±4a | 11±2a | 7±4a | 14±4a | | 10±3a | 10±5a | 50 | |
|  | Gammaproteobacteria | | | | 364±168a | 358±205a | 368±182a | 330±93ab | | 550±57a | 210±49b | 1,308 | |
|  |  | Enterobacteriales | | | 176±190a | 194±244a | 189±249a | 73±28b | | 448±49a | 38±13b | 656 | |
|  |  | Pseudomonadales | | | 95±75a | 65±47a | 96±53a | 139±37a | | 39±2b | 79±52b | 302 | |
|  |  | Others | |  | 93±50a | 99±24a | 82±17a | 118±33a | | 64±10a | 93±13a | 350 | |
| Spirochaetes | |  | |  | 18±6a | 12±5a | 13±5a | 19±4a | | 9±2a | 16±4a | 43 | |
| Tenericutes | |  | |  | 52±24a | 44±5a | 39±3a | 55±23a | | 41±3a | 40±1a | 116 | |
| Verrucomicrobia | | | |  | 16±5a | 15±4a | 15±2a | 15±5a | | 15±4a | 16±2a | 45 | |
| Other |  |  | |  | 82±25a | 80±20a | 69±4a | 95±21a | | 68±4a | 67±3a | 213 | |
| Total |  |  | |  | 2487±1035a | 2383±230a | 2171±695a | 2948±685a | 1981±122a | | 2112±219a | | 7,028 |

PS: 5 g of Penicillin G potassium + 0.5 g of Streptomycin per tree; KO: 2 g of Oxytetracycline + 1.0 g of Kasugamycin per tree; CK: water as control

^y^ The values are the mean±one standard error of the mean (n=3). A statistical analysis was performed using analysis of variance for each phylum, and statistical significance is indicated for a *P* value of 0.05 with different lower-case letters, or for a *P* value of 0.01 with different capital letters. No statistical analysis was performed for phyla containing fewer than five taxa (ND). The factors used in the analyses of variance include antibiotic treatments and time point of sampling.

^Z^ OTUs were detected in at least one of the tested samples.
